# Does Serum Uric Acid Status Influence the Association Between Left Atrium Diameter and Atrial Fibrillation in Hypertension Patients?

**DOI:** 10.3389/fcvm.2020.594788

**Published:** 2020-11-27

**Authors:** Tesfaldet H. Hidru, Yuqi Tang, Fei Liu, Simei Hui, Ruiyuan Gao, Daobo Li, Xiaolei Yang, Yunlong Xia

**Affiliations:** Department of Cardiology, First Affiliated Hospital of Dalian Medical University, Dalian, China

**Keywords:** uric acid, atrial fibrillation, hypertension, hyperuicemia, left atrial diameter (LAD)

## Abstract

**Objective:** Both serum uric acid (SUA) levels and left atrium diameter (LAD) associate with AF. However, the influence of SUA status for the associated risk of AF related to LAD in hypertension patients is currently unknown.

**Methods:** We retrospectively analyzed a hospital-based sample of 9,618 hypertension patients. Standard electrocardiograms were performed on all patients and were interpreted by expert electro-physiologists.

**Results:** Overall 1,028 (10.69%) patients had AF out of 9,618 patients. In men >65 years of age, the prevalence of AF in the1st, 2nd, and 3rd tertiles of SUA among those grouped in the third tertile of LAD were 9, 12.3, and 21.7%, respectively. In the hyperuricemia group, the OR (95% CI) of AF for the highest tertile of LAD in men ≤ 65 years of age was 3.150 (1.756, 5.651; *P* < 0.001). Similarly, the hyperuricemic men in the 3rd LAD tertile had a higher likelihood of AF than those belonging to the 1st tertile. The ORs and (95% CIs) were 3.150 (1.756, 5.651; *P* < 0.001) and 5.522 (2.932, 10.400; *P* ≤ 0.001) for patients ≤ 65 and >65 years of age. An increase in SUA values was significantly associated with an increased likelihood of AF among women at the top tertiles of LAD, with the OR (95% CI) = 4.593 (1.857, 11.358; *P* = 0.001). Also, men> 65 years of age with large LAD, present at the third tertile of SUA, had a higher likelihood of AF, with the OR (95% CI) = 2.427 (1.039, 5.667; *P* < 0.05).

**Conclusion:** SUA levels and LAD are associated with AF in patients with hypertension and the risk of AF associated with LAD increases among those with hyperuricemia.

## Introduction

Atrial fibrillation (AF) is the most sustained arrhythmia, contributing to short and long-term cardiovascular complications such as hemodynamic instability, stroke, heart failure, and mortality risk (1–5). Considering the continuous rise in the average life expectancy in recent years and an increase in cardiac morbidity, the occurrence of AF has been increasing sharply in the past two decades. Despite advancements in the detection and management of AF, inadequate guidance persists, regarding primary prevention and risk stratification of this disease (6).

Several risk factors have been assumed to involve in the pathophysiology of atrial fibrillation, such as hyperuricemia, left atrial diameter (LAD), gender, high-sensitivity C-reactive protein, cystatin-C, obesity, and diabetes (7–9). Of those hypothesized risk factors, increased focus has been given to the possible mechanism by which hyperuricemia causes AF. As such, earlier studies have revealed that elevated serum uric acid (SUA) plays a role in the development of AF in the general population (9–12), as well as in patients with hypertension (13).

AF and hypertension often coexist, and AF patients who experience elevated systolic blood pressure experience increased adverse events (14). Moreover, left atrium volume, diameter, and strain were reported to correlate with new-onset AF in patients suffering from hypertrophic cardiomyopathy (15). Importantly, a piece of evidence also revealed that left atrium enlargement is a marker for increased risk of AF (16–20). Considering the direct effect of LAD in the occurrence and maintenance of AF, and the role of elevated SUA and hypertension in modifying the pathophysiology of AF, it is meaningful for the scientific community to analyze the interaction between SUA level and LAD in AF patients. We hypothesized that elevated SUA levels, in combination with widened LAD, could significantly estimate the risk of AF in patients with hypertension. Therefore, this study aimed to determine the association between SUA levels and LAD with AF and investigate their interaction among the Chinese population with hypertension.

## Materials and Methods

### Population

**Figure 1:**
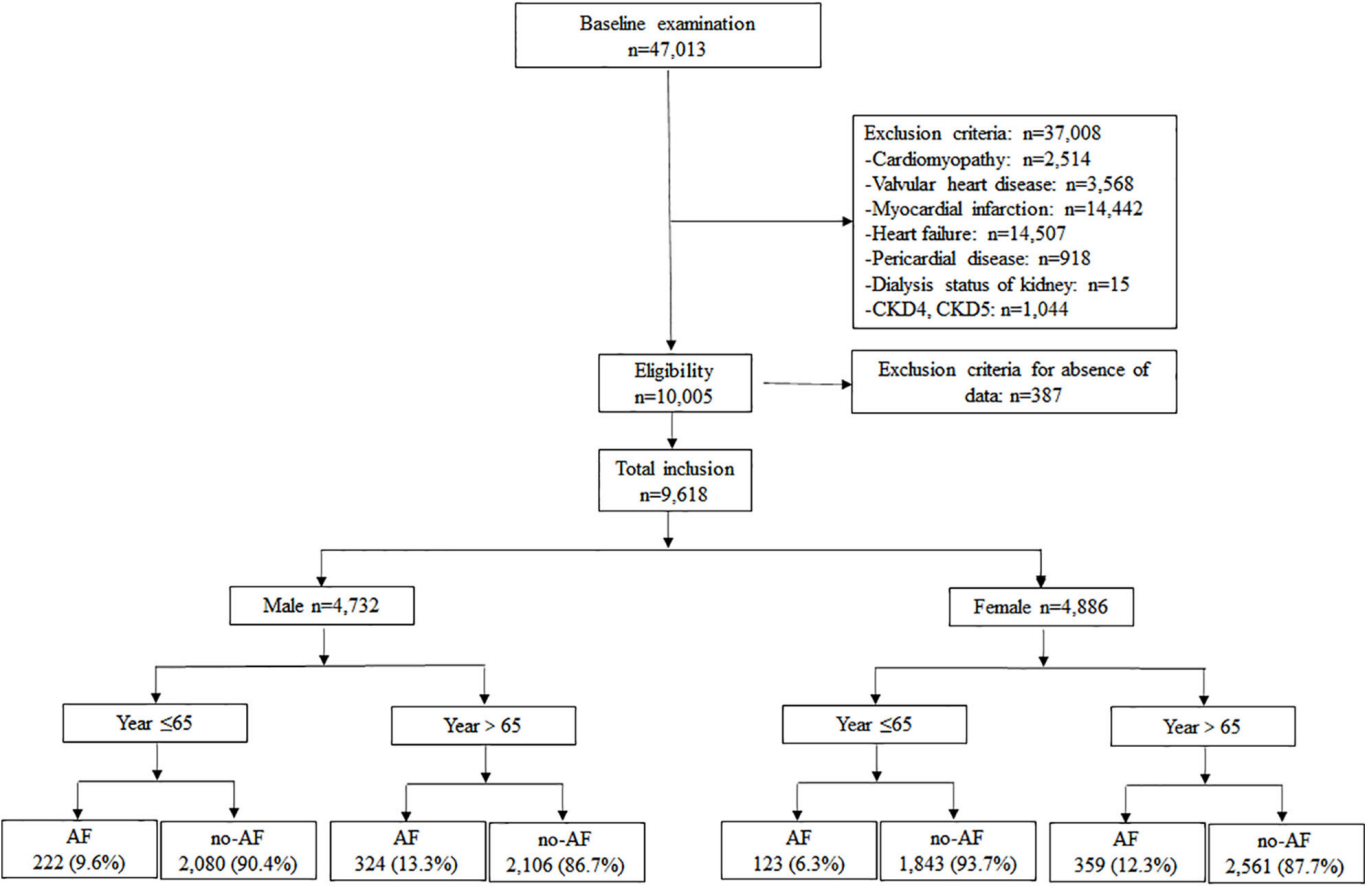
The overview of the selection of study participants.

Hypertension patients, aged 18 to 97 years, hospitalized between August 2015 and August 2018 at the First Affiliated Hospital of Dalian Medical University (FAHDMU) were included. Those with cardiomyopathy, valvular heart disease, myocardial infarction, heart failure, pericardial disease, undergoing dialysis of the kidney, chronic kidney diseases-4 (CKD4) /CKD5, and those patients missing key clinical covariates were excluded. Finally, the present study contained a total of 9,618 patients. Figure 1 describes a brief overview of the selection of study participants. The research was conducted in accordance with the Helsinki declaration guidelines and was approved by the institutional review board of the FAHDMU. The informed consent provision was waived and all procedures listed here were carried out in compliance with the approved guidelines.

### Clinical Measurements and Definition of Explanatory Variables

Demographic and clinical characteristics including age, gender, and major risk factors of hypertension including dyslipidemia, diabetes mellitus, arterial hypertension, alcohol, smoking, and other CVD comorbidities were ascertained from electronic health records. A sample of fasting blood from the brachial vein had been collected. The SUA concentrations were determined using an autoanalyzer using the Uricase-Peroxidase process (BECKMAN COULTER AU680 Chemistry Analyzer, USA). We performed comprehensive 2D transthoracic echocardiography for each patient. All the measurements, including fasting glucose level, serum concentrations of triglycerides, total cholesterol (TC), high-density lipoprotein (HDL) cholesterol, and low-density lipoprotein (LDL) cholesterol were performed at the FAHDMU laboratory using the standard protocols. Hypertension has been characterized as systolic blood pressure (SBP) ≥ 140 mmHg and/or diastolic blood pressure (DBP) ≥ 90 mm Hg or a self-reported history of hypertension with the active use of antihypertensive drugs. Diabetes mellitus (DM) has been defined as fasting 7.0 mmol/L plasma glucose or a self-reported history of diabetes mellitus and/or currently receiving antidiabetic treatments. Dyslipidemia was characterized as TC >240 mg/ dL or LDL cholesterol >160 mg/dL or HDL cholesterol >40 mg/dL and/or lipid-lowering drug use (21). Participants were deemed current smokers if reported they are currently smoking or registered smoking at least 100 cigarettes during their lifetime (22, 23). The approximate glomerular filtration rate (eGFR) was determined using the Renal Disease equation for Diet Modification (24). We measured SUA and LAD when the AF incident was first diagnosed during hospitalization.

### Identification of AF

All subjects received echocardiographic examinations at rest in the left lateral decubitus position using the Vivid 7 ultrasound system (GE Vingmed Ultrasound, Horten, Norway). The Left atrium diameter was obtained based on the American Society of Echocardiography guidelines, a widely used approach to evaluate LAD (25). The LAD was assessed from a parasternal long-axis view at the end-systole (when the LA chamber is at its greatest dimension). Experienced radiologists who were blinded to the clinical data reviewed the echocardiography results.

### Echocardiographic Assessment

All subjects received echocardiographic examinations at rest in the left lateral decubitus position using the Vivid 7 ultrasound system (GE Vingmed Ultrasound, Horten, Norway). The Left atrium diameter was obtained based on the American Society of Echocardiography guidelines, a widely used approach to evaluate LAD (26). The LAD was assessed from a parasternal long-axis view at the end-systole (when the LA chamber is at its greatest dimension). Experienced radiologists who were blinded to the clinical data reviewed the echocardiography results.

### Statistical Analysis

All statistical analyses were conducted using SPSS version 21. Patients were categorized based on age into two groups including, under 65 years of age and above 65 years of age. SUA levels and LAD were stratified into tertiles (T) separately for men and women for the two groups (≤65 and >65 years). The respective cut-off of SUA and LAD values for T1, T2, and T3 for men and women are given in the footnote of each table. All categorical variables were expressed as counts and percentiles and continuous variables were expressed as mean ± SD. Variables were compared for differences between AF and non-AF patients using two independent sample *t*-test and χ^2^ test for continuous and categorical data, respectively. Binary logistic regression models were estimated, and the odds ratios (OR) at 95% confidence interval (CIs) were used to approximate the associated risk for AF according to tertiles of SUA levels and LAD, with the lowest tertile serving as the reference category. Model 1 was adjusted for age. Model 2 was adjusted for age, SBP, serum creatinine, and smoking. Model 3 was adjusted for the covariates in model 2, followed by dyslipidemia and DM, statin, and antihypertensive agents. Further, we ran a sub-analysis to estimate the associated AF risk across the tertiles of LAD in hyperuricemic and normouricemic patients, and the associated AF risk across tertiles of SUA in patients with normal and widened LAD. All statistical analyses were two-sided, and a *P* < 0.05 was considered statistically significant.

## Results

### Baseline Characteristics of the Participants

Among the 47,013 patients who were included, a total of 9,618 patients (4,732 men and 4,886 women) were selected in the final analysis. The mean ± SD ages of AF and non-AF patients were 69.51 ± 9.20 and 66.30 ± 10.49, respectively. In total, 546 of the 4,732 men (11.54%) had AF, which accounted for 53.11% of the AF population. In the entire population, the AF patients had a higher mean age, SUA, and LAD than the non-AF group. However, patients with AF had a lower mean SBP compared with non-AF patients. The proportion of patients, who were in diuretic and β-blocker use, was higher in AF patients compared to the non-AF group (Table 1).

**Table 1:** Baseline characteristics of the participants.

**Variables**	**AF (*n* = 1,028)**	**no-AF (*n* = 8,590)**	***P*-value**
Male (*n*/%)	546 (53.1%)	4,186 (48.7%)	0.008
Age (year)	69.51 ± 9.20	66.30 ± 10.49	<0.001
**Blood pressure readings**			
SBP (mm Hg)	141.54 ± 19.50	146.29 ± 20.96	<0.001
DBP (mm Hg)	82.65 ± 13.31	82.90 ± 12.81	0.566
DM (*n*/%)	264 (25.7%)	2,502 (29.1%)	0.021
Dyslipidemia (*n*/%)	793 (77.1%)	7,078 (82.4%)	<0.001
**Lipid panel**			
TC (mg/dL)	179.67 ± 40.27	186.28 ± 42.50	<0.001
TG (mg/dL)	132.70 ± 74.56	149.18 ± 102.96	<0.001
HDL (mg/dL)	47.25 ± 10.47	47.25 ± 11.24	0.977
LDL (mg/dL)	104.96 ± 28.97	104.97 ± 30.24	0.995
Smoking (*n*/%)	219 (21.6%)	1,989 (24.6%)	0.037
Alcohol (*n*/%)	145 (14.6%)	1,242 (15.7%)	0.351
SUA (mol/L)	356.59 ± 90.99	340.73 ± 89.63	<0.001
Scr (umol/L)	75.24 ± 42.02	70.59 ± 35.85	<0.001
LAEDD (mm)	39.57 ± 5.05	36.58 ± 3.42	<0.001
**Antihypertensive agent**			
ACEI/ARB (*n*/%)	569 (55.4%)	4,779 (55.6%)	0.862
β-blocker (*n*/%)	675 (65.7%)	4,211 (49.0%)	<0.001
CCB (*n*/%)	625 (60.8%)	5,490 (63.9%)	0.500
Statin (*n*/%)	719 (69.9%)	6,567 (76.4%)	<0.001
Diuretic (*n*/%)	179 (17.45)	1,197 (13.9%)	0.003

*AF, Atrial fibrillation; SBP, Systolic blood pressure; DBP, Diastolic blood pressure; DM, Diabetes mellitus; TC, Total Cholesterol TG, triglyceride; HDL, high-density lipoprotein; LDL, low-density lipoprotein; SUA, Serum uric acid; Scr, Serum creatinine; LAEDD, Left atrial end-diastolic diameter; ACEI, Angiotensin-Converting Enzyme Inhibitors; ARB, angiotensin-converting enzyme receptor blockers; and CCB, Calcium channel blockers*.

Unlike younger female patients, male patients under 65 years of age in the AF group were more likely to have DM and dyslipidemia. Conversely, women patients who were above 65 years of age, were more likely to have DM and dyslipidemia. Regardless of the age group, patients in the AF group were more likely to use β-blockers. Demographic data for the patients in the two groups separately are shown for men and women in Supplementary Tables 1, 2, respectively.

### The Prevalence of AF

Overall, 1,028 (10.69%) patients had AF out of 9618 patients. 222/2302 AF occurred in men patients who were under 65 years of age, whereas 324/2430 AF occurred in male subjects over 65 years of age. In the group of ≤ 65 years of age, the prevalence of AF was 9.6% in men and 6.3% in women.

The prevalence of AF in patients ≤ 65 years of age, categorized at the 3rd SUA tertile, was 10.5% compared with the prevalence of AF in those belonging to 2nd and 1st tertile (8.7 and 5.1%, respectively). In the group of ≤ 65 years of age, the prevalence of AF was significantly increased from 3.5% in the low tertile to 6.7 and 14.0% in the middle and high tertiles of LAD, respectively. Similarly, the prevalence of AF in old aged patients was significantly increased from 7.8% in the low to 10.1 and 22.9% in the middle and high tertiles of LAD, respectively.

### Relationship Between SUA/LAD and AF

The association between the SUA levels and the risk of AF among patients above 65 years old is presented in Table 2. When the values of SUA were treated as continuous data, the adjusted OR and 95% CI for AF in men ≤ 65 years of age was 1.002(1.010, 1.004, *P* = 0.001). This association persisted when SUA values were divided into three tertiles. In the ≤ 65-year-old group, the third tertile of SUA concentrations was significantly associated with AF in both men and women. In the fully adjusted multivariate analysis, the ORs and 95% CIs of AF for the patients in tertile 3 compared to patients in the first tertile of SUA were 2.098 (1.393, 3.161; *P* < 0.001) and 1.805 (1.070, 3.044; *P* < 0.05), respectively.

**Table 2:** The relationship between SUA/LAD and Atrial fibrillation.

**Men**	**Unadjusted model**				**Adjusted model**			
	**Age** **≤65 (*****n*** **=** **2,302)**		**Age** **>65 (*****n*** **=** **2,430)**		**Age** **≤65 (*****n*** **=** **2,302)**		**Age** **>65 (*****n*** **=** **2,430)**	
	**OR (95% CI)**	***P*-value**	**OR (95% CI)**	***P*-value**	**OR (95% CI)**	***P*-value**	**OR (95% CI)**	***P*-value**
SUA	1.002 (1.001, 1.004)	0.001	1.020 (1.010, 1.040)	0.017	1.003 (1.001, 1.004)	0.003	1.000 (0.998, 1.001)	0.934
LAEDD	1.229 (1.188, 1.273)	<0.001	1.177 (1.143, 1.212)	<0.001	1.25 (1.206, 1.304)	<0.001	1.189 (1.152, 1.226)	<0.001
**Women**	**Age** **≤65 (*****n*** **=** **1,966)**		**Age** **>65 (*****n*** **=** **2,920)**		**Age** **≤65 (*****n*** **=** **1,966)**		**Age** **>65 (*****n*** **=** **2,920)**	
	**OR (95% CI)**	***P*****-value**	**OR (95% CI)**	***P*****-value**	**OR (95% CI)**	***P*****-value**	**OR (95% CI)**	***P*****-value**
SUA	1.003 (1.001, 1.005)	0.007	1.001 (1.000, 1.002)	0.090	1.001 (0.999, 1.004)	0.290	1.000 (0.998, 1.001)	0.778
LAEDD	1.227 (1.166, 1.293)	<0.001	1.208 (1.173, 1.245)	<0.001	1.195 (1.134, 1.260)	<0.001	1.212 (1.173, 1.252)	<0.001

*Adjusted for age, systolic blood pressure, serum creatinine, smoking, dyslipidemia and diabetes mellitus, statin, ACEI, ARB, CCB, β-blocker*.

The result of this study also shows a positive association between the higher tertiles of LAD and the presence of AF in men and women in both age groups after adjusting for age, SBP, serum creatinine, and smoking (Supplementary Table 3). In the men ≤ 65-year-old group, compared with the first tertile of LAD, the multivariate-adjusted OR and 95% CI of AF for the second and third tertiles were 1.581 (1.029, 2.429); *P* < 0.05), and 4.473 (2.991, 6.688; *P* < 0.001), respectively. In the group of >65 years, the OR associated with AF in those belonging to the third tertile of LAD was increased by nearly four-folds compared to the first tertile after adjustment for multiple confounding variables (adjusted OR = 3.992, 95% CI: 2.871, 5.549; *P* < 0.001). Table 3 presents the prevalence and ORs of AF among men grouped by the tertiles of SUA levels and LAD.

**Table 3:** The prevalence of AF and the risk estimate for the atrial fibrillation based on the tertiles of SUA/LAD.

	**Age** **≤65 (*****n*** **=** **4,268)**			**Age** **>65 (*****n*** **=** **5,350)**		
**Tertiles of serum uric acid levels**						
	T1 (*n* = 1,428)	T2 (*n* = 1,417)	T3 (*n* = 1,423)	T1 (*n* = 1,785)	T2 (*n* = 1,799)	T3 (*n* = 1,766)
No. of AF(%)	73 (5.1%)	123 (8.7%)	149 (10.5%)	198 (11.1%)	241 (13.4%)	244 (13.8%)
	**OR (95% CI)**	**OR (95% CI)**	**OR (95% CI)**	**OR (95% CI)**	**OR (95% CI)**	**OR (95% CI)**
Men	Ref.	1.892 (1.267, 2.825)^†^	2.098 (1.393, 3.161)^‡^	Ref.	1.044 (0.764, 1.426)	1.061 (0.774, 1.454)
Women	Ref	1.546 (0.923, 2.591)	1.805 (1.070, 3.044)^*^	Ref.	0.868 (0.642, 1.172)	1.009 (0.753, 1.353)
**Tertiles of LAD**						
	T1 (*n* = 1,421)	T2 (*n* = 1,425)	T3 (*n* = 1,422)	T1 (*n* = 2,109)	T2 (*n* = 1,754)	T3 (*n* = 1,487)
No. of AF(%)	50 (3.5%)	96 (6.7%)	199 (14.0%)	165 (7.8%)	177 (10.1%)	341 (22.9%)
	**OR (95% CI)**	**OR (95% CI)**	**OR (95% CI)**	**OR (95% CI)**	**OR (95% CI)**	**OR (95% CI)**
Men	Ref.	1.581 (1.029, 2.429)^*^	4.473 (2.991, 6.688)^‡^	Ref.	1.713 (1.201, 2.444)^†^	3.992 (2.871, 5.549)^‡^
Women	Ref	1.136 (0.647, 1.997)	2.628 (1.627, 4.243)^‡^	Ref.	1.056 (0.755, 1.479)	3.178 (2.366, 4.269)^‡^

* *P < 0.05*,

† *P < 0.01*,

‡ *P < 0.001*.

*Age ≤ 65 men = Tertile 1, ≤303.00 μmol/L; SUA-Tertile 2, 303.00–376.93 μmol/L, and SUA-Tertile 3, >376.93 μmol/L; LAEDD-Tertile 1, ≤35.90 mm, LAEDD-Tertile 2, 35.90–37.26 mm, and LAEDD-Tertile 3, >37.26 mm. Age >65 men = SUA-Tertile 1, ≤298.00 μmol/L, SUA-Tertile 2, 298.00–366.00 μmol/L, and SUA-Tertile 3,>366.00 μmol/L; LAEDD-Tertile 1, ≤36.00 mm, LAEDD-Tertile 2, 36.00–38.00 mm, and LAEDD-Tertile 3, >38.00 mm. Adjusted for age, SBP, Serum creatinine, smoking, dyslipidemia and DM, statin, diuretic, ACEI, ARB, CCB, β-blocker*.

Women in the third tertile had a higher AF prevalence compared with those in the first and second LAD tertiles. This relationship persisted even after adjusting for potential confounders including, age, SBP, serum creatinine, smoking, dyslipidemia, DM, statin use, and antihypertensive agents such as ACEI, ARB, CCB, and β-blocker use. In patients ≤65 years of age, the OR (95% CI) of AF for the women in T3 compared to the first tertile of LAD was 2.628 (1.627, 4.243; *P* < 0.001). Among women older than 65 years of age, the third tertile of LAD was associated with a nearly three-fold increased risk of AF compared to those in the first tertile of LAD after adjustment for multiple confounding factors [the adjusted OR (95% CI) = 3.178 (2.366, 4.269; *P* < 0.001)].

### The Effect of SUA and LAD Interaction in AF

**Figure 2:**
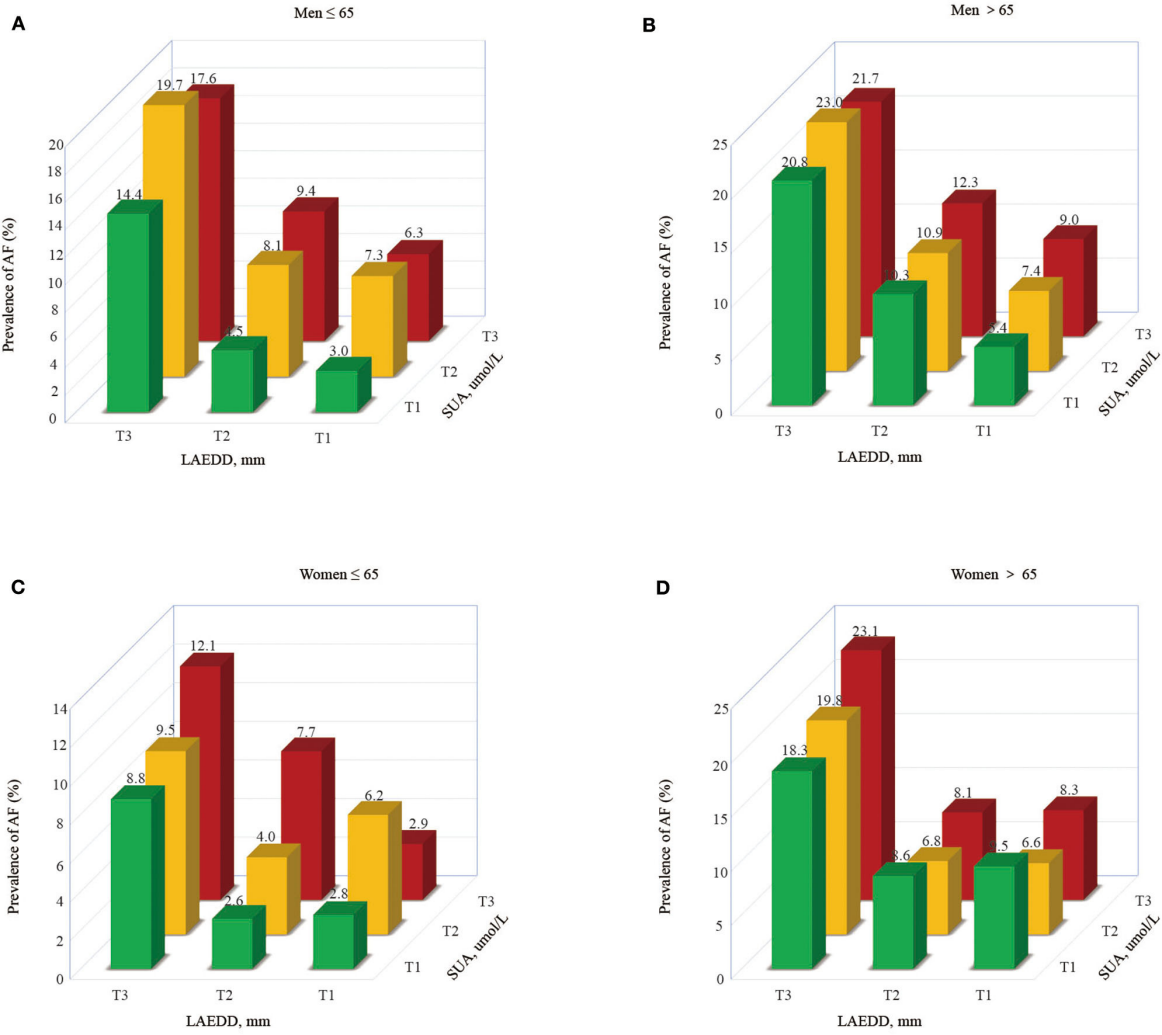
The prevalence of AF based on LAD tertiles in patients grouped by SUA tertiles.

To compute the interaction effect of elevated SUA and enlarged LAD, we calculated the prevalence of AF and estimated the OR and 95% CI of AF among those patients grouped in different tertiles of SUA along their corresponding LAD tertiles. The prevalence of AF increases across SUA and LAD tertiles, implying the patients in higher tertiles of SUA and LAD had a higher prevalence of AF than in lower tertiles. An increase in LAD shows a progressively higher prevalence of AF across the tertiles of SUA (Figure 2). With an increase in tertiles of LAD, the men under the age of 65 years had a higher prevalence of AF across the tertiles of SUA (6.35 vs. 9.4 vs. 17.6%, respectively). Moreover, those men >65 years in the 3rd tertile of SUA grouped by LAD tertiles had a higher prevalence of AF than those belonging to 2nd or 1st tertile (9, 12.3, and 21.7% for the 1st, 2nd, and 3rd tertiles of SUA, respectively). The prevalence of AF in the1st, 2nd, and 3rd tertiles of SUA for those women >65 years of age grouped in the third tertile of LAD were 18.3, 19.8, and 23.1%, respectively.

**Figure 3:**
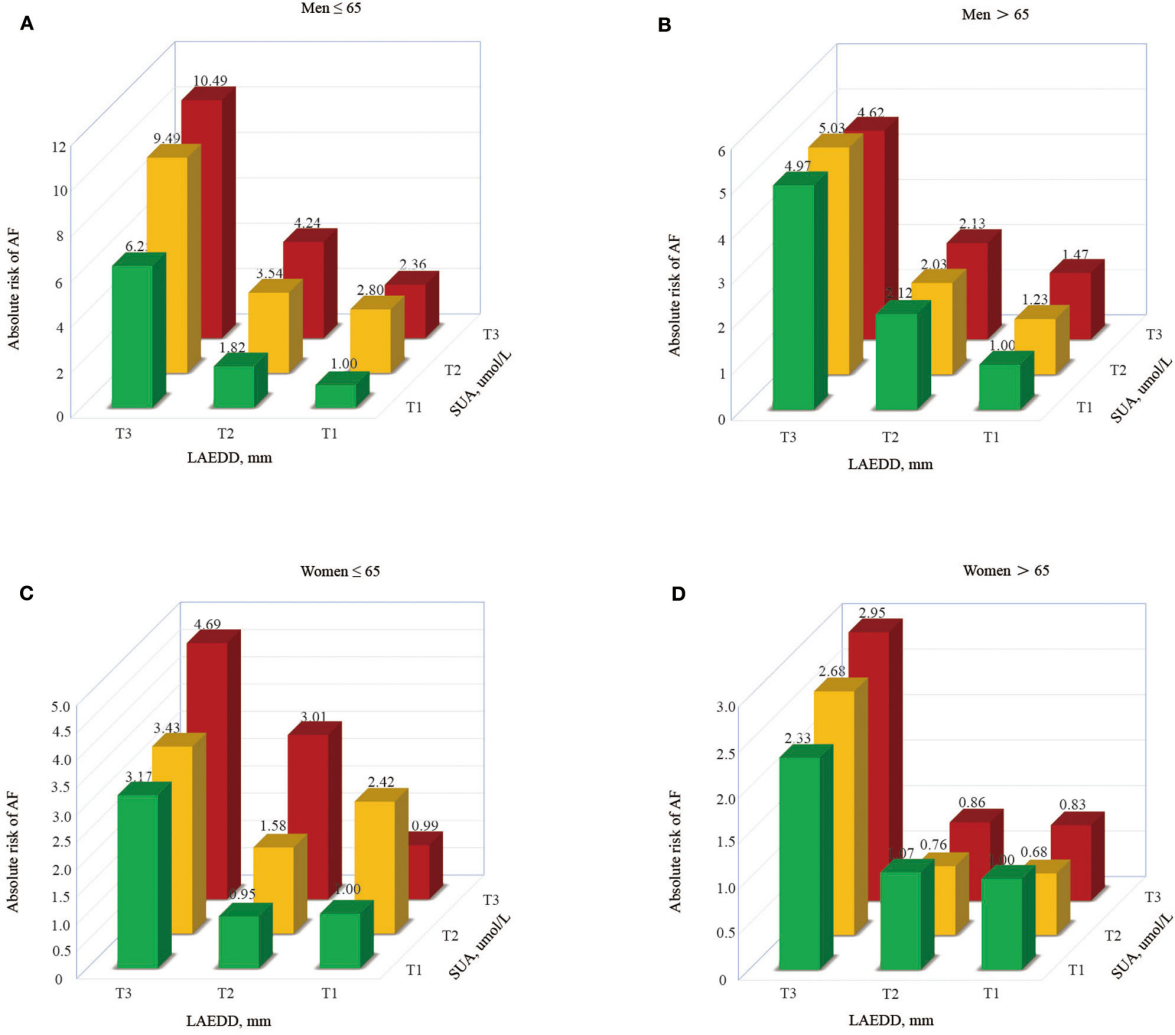
The risk of AF based on LAD tertiles in patients grouped by SUA tertiles.

Those patients at higher tertiles of SUA grouped by LAD had a higher risk of AF, with patients in T3 accounting for the highest risk of AF (OR = 10.49 in men ≤65 and 4.62 in men>65, respectively). Similarly, the estimated risk of AF in young aged women was significantly increased from 3.17 to 4.69% across the first to third tertile of LAD (in those patients grouped by SUA tertiles), respectively. At the same time, the women at higher tertiles of LAD grouped by SUA had a higher risk of AF in the old aged population, with patients in T3 accounting for the highest risk of AF (OR = 2.33, 2.68, and 2.95 for T1, T2, and T3, respectively). Figure 3 describes the risk of AF based on LAD tertiles in patients grouped by SUA tertiles.

Table 4 presents the odds ratios associated with an increase in SUA among participants grouped by tertiles of LAD. With an increase in SUA levels, the regression analysis confirmed that men under the age of 65 years in the third tertile of LAD had an independent increase in risk for AF. The ORs (95% CIs) for the 1st, 2nd, and 3rd tertiles of SUA were 3.403 (1.787, 6.478), 4.596 (2.355, 8.968), and 6.614 (2.870, 15.245), respectively. Similarly, an increase in SUA values in women was significantly associated with an increased likelihood of AF among those of the highest tertiles of LAD, with the OR (95% CI) = 4.593 (1.857, 11.358; *P* = 0.001). Also, in the population above 65 years (both men and women), an increase in SUA values was markedly associated with an increased risk for AF among those of the highest tertiles of LAD, suggesting a substantial AF risk was present among those patients classified at the highest tertile of SUA levels and LAD.

**Table 4:** The prevalence of AF according to baseline SUA tertile grouped by LAD in men and women.

	**Age** **≤65 Years**					**Age** **>65 Years**				
**SUA**	**T1 (*N* = 772)**	**T2 (*****N*** **=** **842)**		**T3 (*****N*** **=** **688)**		**T1 (*N* = 818)**	**T2 (*****N*** **=** **802)**		**T3 (*****N*** **=** **810)**	
		**OR (95% CI)**	***P*****-value**	**OR (95% CI)**	***P*****-value**		**OR (95% CI)**	***P*****-value**	**OR (95% CI)**	***P*****-value**
**Tertile of LAD in men (*****N*** **=** **4,732)**										
T1	Ref.	1.812 (0.747, 4.398)	0.189	3.403 (1.787, 6.478)	<0.001	Ref.	2.188 (1.156, 4.141)	0.016	3.188 (1.867, 5.441)	<0.001
T2	Ref.	1.282 (0.654, 2.514)	0.469	4.596 (2.355,8.968)	<0.001	Ref.	1.624 (0.876, 3.011)	0.124	4.082 (2.297, 7.255)	<0.001
T3	Ref.	1.839 (0.888, 3.806)	0.101	6.614 (2.870, 15.245)	<0.001	Ref.	1.450 (0.790, 2.664)	0.231	4.790 (2.586, 8.872)	<0.001
	**Age** **≤65 Years**					**Age** **>65 Years**				
	**T1 (*****n*** **=** **712)**	**T2 (*****n*** **=** **599)**		**T3 (*****n*** **=** **655)**		**T1 (*****n*** **=** **973)**	**T2 (*****n*** **=** **973)**		**T3 (*****n*** **=** **974)**	
**SUA**		**OR (95% CI)**	***P*****-value**	**OR (95% CI)**	***P*****-value**		**OR (95% CI)**	***P-*****value**	**OR (95% CI)**	***P-*****value**
**Tertile of LAD in women (*****N*** **=** **4,868)**										
T1	Ref.	0.862 (0.279, 2.658)	0.796	3.347 (1.244, 9.006)	0.017	Ref.	1.054 (0.618, 1.798)	0.846	2.331 (1.412, 3.847)	0.001
T2	Ref.	0.604 (0.240, 1.521)	0.285	1.488 (0.674, 3.289)	0.326	Ref.	1.164 (0.621, 2.182)	0.637	3.622 (2.190, 5.991)	<0.001
T3	Ref.	3.577 (1.233, 10.372)	0.019	4.593 (1.857, 11.358)	0.001	Ref.	1.037 (0.557, 1.931)	0.909	4.210 (2.364, 7.495)	<0.001

*Adjusted for age, SBP, Serum creatinine, smoking, dyslipidemia, DM, statin, diuretic, ACEI, ARB, CCB, and β-blocker*.

### The Impact of Left Atrium End-Diastolic Diameter in Atrial Fibrillation Patients With Normouricemia and Hyperuricemia

To investigate whether there was a possibility that the hyperuricemic status might have influenced the predictive power of LAD values for AF among the patients with hypertension, we calculated the multivariable-adjusted ORs and 95% CIs in two separate groups, normouricemia (*n* = 7,196) and hyperuricemic (*n* = 2,422). Those men with a large LAD value, regardless of the hyperuricemic status, had a greater likelihood of having AF. The OR (95% CI) of AF for the highest tertile of LAD was 3.150 (1.756, 5.651; *P* < 0.001) in hyperuricemic middle-aged men. Likewise, those hyperuricemic old aged men in the 3rd tertile of LAD had a higher likelihood of AF than those belonging to the 1st tertile, the OR (95% CI) was 5.522 (2.932, 10.400; *P* ≤ 0.001). Similar findings were observed in the normouricemic group. Compared to the first tertile of SUA, the OR and 95% CI of AF for men younger and older than 65 years of age in the highest tertile were 4.976 (3.044, 8.136: *P* < 0.001) and 4.150 (2.832, 6.081; *P* < 0.001), respectively. In the group of >65 years of age, the risk of AF was significantly increased in the third tertiles of LAD in both hyperuricemic and normouricemic women (OR = 2.947 and 4.336, respectively). The impact of LAD in atrial fibrillation in patients with normouricemia and hyperuricemia is summarized in Table 5.

**Table 5:** The impact of left atrium diameter in atrial fibrillation patients with normouricemia and hyperuricemia.

**Men**	**Age** **≤65**				**Age** **>65**			
**Tertiles of LAD**								
	**T1 (*****n*** **=** **772)**	**T2 (*****n*** **=** **842)**	**T3 (*****n*** **=** **688)**		**T1 (*****n*** **=** **818)**	**T2 (*****N*** **=** **802)**	**T3 (*****n*** **=** **810)**	
**AF (*****n*****, %)**	41 (5.3%)	61 (7.2%)	120 (17.4%)		58 (7.1%)	89 (11.1%)	177 (21.9%)	
	**OR (95% CI)**	**OR (95% CI)**	**OR (95% CI)**	***P-trend***	Ref.	**OR (95% CI)**	**OR (95% CI)**	***P-trend***
Normouricemia	Ref.	1.532 (0.908, 2.586)	4.976 (3.044, 8.136)^‡^	<0.001	Ref.	1.824 (1.217, 2.735)^†^	4.150 (2.832, 6.081)^‡^	<0.001
Hyperuricemia	Ref.	1.049 (0.502, 2.193)	3.150 (1.756, 5.651)^‡^	<0.001	Ref.	1.521 (0.765, 3.023)	5.522 (2.932, 10.400)^‡^	<0.001
**Women**	**T1 (*****n*** **=** **712)**	**T2 (*****n*** **=** **599)**	**T3 (*****n*** **=** **655)**		**T1 (*****n*** **=** **973)**	**T2 (*****n*** **=** **973)**	**T3 (*****n*** **=9 74)**	
**AF (n, %)**	28 (3.9%)	26 (4.3%)	69 (10.5%)		80 (8.2%)	76 (7.8%)	203 (20.8%)	
	**OR (95% CI)**	**OR (95% CI)**	**OR (95% CI)**	***P-trend***		**OR (95% CI)**	**OR (95% CI)**	***P-trend***
Normouricemia	Ref.	1.075 (0.552, 2.094)	2.593 (1.491, 4.511)^†^	0.001	Ref.	1.015 (0.689, 1.496)	2.947 (2.094, 4.147)^‡^	<0.001
Hyperuricemia	Ref.	1.125 (0.416, 3.045)	1.668 (0.720, 3.863)	0.227	Ref.	1.190 (0.638, 2.217)	4.336 (2.488, 7.557)^‡^	<0.001

† *P < 0.01*,

‡ *P < 0.001*.

*In men: ≤ 420 μmol/L considered normal SUA levels; and >420 μmol/L level was considered as hyperuricemia. In women: Normal: ≤ 360 μmol/L and High: >360 μmol/L. Men Age ≤65 = LAD-Tertile 1, ≤36.00 mm. LAEDD-Tertile 2, 36.00-38.00 mm and LAD-Tertile 3, >38.00 mm. Men Age >65 = LAEDD-Tertile 1, ≤36.00 mm, LAD-Tertile 2, 36.00-38.09 mm, and LAEDD-Tertile 3, >38.09 mm. Women Age ≤65 = LAEDD-Tertile 1, ≤35.00 mm, LAD-Tertile 2, 35.00-36.82 mm; and LAD-Tertile 3, >36.82 mm. Women Age >65 = LAD-Tertile 1, ≤35.69 mm; LAD-Tertile 2, 35.69-37.36 mm; LAEDD-Tertile 3, >37.36 mm. Adjusted for age, SBP, Scr, smoking, dyslipidemia, DM, statin, diuretic, ACEI, ARB, CCB, and β-blocker*.

### The Impact of Serum Uric Acid in Atrial Fibrillation in Patients With Normal and Enlarged Left Atrium Diameter

We performed sub-analyses by applying recently published LAD criteria (27). The left atrium was considered enlarged when left atrial diameter exceeded 4.2 cm in men and 3.8 cm in women, thus the patients were grouped into two categories based on their LAD size: normal LAD ≤4.2 cm and large LAD >4.2 cm in men, and normal LAD ≤3.8 cm and large LAD >3.8 cm in women. When patients were broken down based on the size of LAD, elevated levels of SUA was associated with greater odds of AF in those patients with normal LAD in the younger age group. The effect of SUA appeared most pronounced among men diagnosed with AF categorized under the age group of ≤65 years of age. The ORs (95% CI) for the middle and the highest tertiles of SUA compared to the lowest tertile were 2.139 [1.361, 3.361; *P* < 0.01) and 2.228 (1.397, 3.552; *P* = 0.01)], respectively. Moreover, those patients >65 years of age with large LAD, present at the third tertile of SUA, had a higher likelihood of AF, with the OR (95% CI) = 2.427 (1.039, 5.667; *P* < 0.05). The AF risk was lower in the normal LAD group in women above 65 years of age compared with those patients in the first tertile [Adjusted OR (95% CI) = 0.656 (0.446, 0.966; *P* < 0.05]. However, there was no significant risk of AF associated with an increase in SUA in the normal LAD and large LAD groups in the women population. Table 6 presents the impact of SUA in AF among hypertension patients with normal and enlarged LAD.

**Table 6:** The impact of serum uric acid in atrial fibrillation patients with normal and wide left atrium diameter.

	**Age** **≤65**				**Age** **>65**			
**Tertiles of SUA**								
**Men**	**T1 (*****n*** **=** **767)**	**T2 (*****n*** **=** **771)**	**T3 (*****n*** **=** **764)**		**T1 (*****n*** **=** **818)**	**T2 (*****n*** **=** **803)**	**T3 (*****n*** **=** **809)**	
**AF (*****n*****, %)**	47 (6.1%)	86 (11.2%)	89 (11.6%)		92 (11.2%)	109 (13.6%)	123 (15.2%)	
	**OR (95% CI)**	**OR (95% CI)**	**OR (95% CI)**	***P-trend***	**OR (95% CI)**	**OR (95% CI)**	**OR (95% CI)**	***P-trend***
Normal LAEDD	Ref.	2.139 (1.361, 3.361)^†^	2.228 (1.397, 3.552)^†^	0.001	Ref.	1.116 (0.792, 1.574)	1.094 (0.722, 1.552)	0.639
Large LAEDD	Ref.	1.364 (0.549, 3.389)	2.239 (0.901, 5.567)	0.081	Ref.	1.595 (0.738, 3.450)	2.427 (1.039, 5.667)^*^	0.041
**Women**	**T1 (*****n*** **=** **659)**	**T2 (*****n*** **=** **663)**	**T3 (*****n*** **=** **644)**		**T1 (*****n*** **=** **977)**	**T2 (*****n*** **=** **972)**	**T3 (*****n*** **=** **971)**	
**AF (*****n*****, %)**	27 (4.1%)	43 (6.5%)	53 (8.2%)		113 (11.6%)	103 (10.6%)	143 (14.7%)	
	**OR (95% CI)**	**OR (95% CI)**	**OR (95% CI)**	***P-trend***	**OR (95% CI)**	**OR (95% CI)**	**OR (95% CI)**	***P-trend***
Normal LAEDD	Ref.	1.565 (0.860, 2.848)	1.612 (0.862, 3.014)	0.148	Ref.	0.703 (0.485, 1.019)	0.656 (0.446, 0.966)^*^	0.033
Large LAEDD	Ref.	2.074 (0.850, 5.060)	1.534 (0.590, 3.984)	0.381	Ref.	1.739(1.075, 2.812) ^*^	1.600 (0.964, 2.658)	0.086

* P < 0.05,

† *P < 0.01*.

*In men: ≤42 mm considered normal LAD levels; and >42 mm was considered as left atrial enlargement. In women: ≤38 mm was considered normal LAD and >38 mm was considered as left atrial enlargement. Age ≤65 men: SUA-Tertile 1, ≤337.59 μmol/L; SUA-Tertile 2, 337.59-409.00 μmol/L; SUA-Tertile 3,>409.00 μmol/L. Age >65 men: SUA-Tertile 1, ≤323.00 μmol/L; SUA-Tertile 2, 323.00-393.00 μmol/L; SUA-Tertile 3,>393.00 μmol/L. Age ≤65 Women: SUA-Tertile 1, ≤272.00 μmol/L; SUA-Tertile 2, 272.00-337.00 μmol/L; SUA-Tertile 3, >337.00 μmol/L; Age >65 women: SUA-Tertile 1, ≤279.00 μmol/L; SUA-Tertile 2, 279.00-342.00 μmol/L; SUA-Tertile 3,> 342.00 μmol/L. Adjusted for age, SBP, Scr, smoking, dyslipidemia, DM, statin, diuretic, ACEI, ARB, CCB, and β-blocker*.

## Discussion

In this cross-sectional study, conducted in 9,618 hypertension patients from the hospital registry, elevated SUA and LAD were independently associated with an increased prevalence of AF. Also, the interaction analysis shows that patients in the highest SUA and LAD tertile had a significantly increased risk of AF. This association of the SUA concentrations and LAD with AF remains consistent even after adjusting for potential confounders, which confirmed that SUA levels and LAD could predict the presence of AF.

Hypertension, a well-recognized public health burden worldwide, is associated with an increased risk of AF (28). Furthermore, AF and hypertension often coexist in hyperuricemic patients. For instance, earlier evidence suggested that increased SUA level positively associates with AF prevalence in patients with chronic systolic heart failure (29). A recent study, which enrolled patients aged ≥35 years in the rural Liaoning province of China, proposed an independent association between SUA and AF in the total population and men after adjusting for conventional CVD risk factors (30). Likewise, the present data suggested that elevated SUA was associated with AF in individuals with large LAD. In our study, the prevalence of AF increased from 5.1 to 10.5% across T1-T3 of SUA levels in individuals ≤65-year-old and from 11.1 to 13.8% across T1-T3 of SUA levels in aged patients (>65-year-old). These results demonstrate a substantial increase in the proportion of AF patients with an increase in SUA levels.

According to the present study, the mean LAD was significantly higher in hypertension patients with AF than their counterparts without AF. This finding is in line with the previous observations among the general population (7, 20). Thus, the findings of the present study consolidated the association between the LAD and the presence of AF in hypertension patients. As per our results, those patients with an increased SUA level and LAD had a higher likelihood of AF. It has been previously demonstrated that hyperuricemia contributes to atrial remodeling, large left atrial size (7), ionic channel remodeling, metabolic syndrome (31), endothelial dysfunction (32), and arterial stiffness (33). Conversely, the use of allopurinol, a medication that lowers uric acid via xanthine oxidase inhibition mechanism, is associated with a lower risk of AF (34). Hence, the possible biological explanations for the link between serum urate and risk of AF could be attributed to the mechanism that involves xanthine oxidase-mediated oxidative stress.

Several biological speculations have been suggested for the link between SUA and the risk of AF. Putative mechanisms through which SUA participates in AF development can be summarized as follows: First, an elevated SUA level is indeed an independent marker of various cardiovascular events. In many instances, there is a mutual relationship between SUA and cardiovascular risk factors (insulin resistance, chronic kidney disease conditions, metabolic syndrome, overweight/obesity); and subsequently teasing out the distinct influence of individual factors has proven a challenge to the research community. In this regard, an increased SUA level may be considered as an epiphenomenon of co-existing cardio-metabolic risk or a correlate of cardiovascular risk factors. Second, SUA is a product of xanthine-oxidoreductase activity (XOR), which is known to be one of the most essential courses of reactive oxygen species (ROS) in an organism. XOR *per se* has extensive implications in CVD and is closely interrelated to another key ROS producer, the enzyme NADPH oxidase (35). It is also well-established that XOR activity associates with risk factors for CVD and inflammatory markers (36). Furthermore, SUA may represent an endogenous signal of cell injury activating the cellular immune response. In fact, SUA has been associated with systemic inflammatory markers [such as c-reactive protein (CRP), interleukin (IL)-1, IL-6, IL-8, and tumor necrosis factor-α (TNF-α)] (37). These inflammatory cytokines (IL-6, IL-8, IL-1β, and TNF-α) exert differential effects on vascular inflammation and dysfunction in patients with gout, a condition characterized by hyperuricemia. Additionally, uric acid increases the expression of angiotensin II in vascular endothelial cells (38) and activates the intrarenal renin-angiotensin system in humans (39). Likewise, experimental studies showed that uric acid promotes vasoconstriction and vascular smooth muscle cell proliferation (40), which could further increase the risk of vascular injury (due to inflammation and endothelial damage), vascular resistance, and cardiac hypertrophy. As such, SUA induced inflammation, oxidative stress, and endothelial dysfunction are factors increasing the risk of AF. Third, the electrophysiological hypothesis is also one of the conceivable mechanisms for the SUA induced AF. According to the electrophysiological hypothesis, uric acid enters atrial cells through uric acid transporters and stimulates Kv1.5 protein expression which could further contribute to an increased Kv1.5 ion activity and channel/Ikur current that reduce the action potential duration of atrial cardiomyocytes (41). Overall, the possible explanation for the connection between SUA and AF may support the pathophysiology milieu of vascular function injury and electrophysiologic theory.

The interaction analysis among those patients grouped in the different tertiles of SUA and their corresponding tertiles of LAD demonstrated that most patients at the top tertiles of SUA level and LAD had a higher likelihood of AF. These results provide support for the speculated link between SUA and LAD with the prevalence of AF. Though the underlying mechanisms of elevated SUA relating to the risk of enlarged LAD are poorly understood, some studies intend to elaborate on the mechanism that involves xanthine oxidase-mediated oxidative stress and inflammation. Xanthine oxidase activity alters several important physiological functions, including vessel diameter modulation, remodeling, and lesion formation. The consequences of inflammation and oxidative stress can lead to cardiac remodeling and atrial fibrosis, which may increase the susceptibility of AF. Of note, chronic inflammation is well-known for its contribution to endothelial damage, enhanced activity of the platelet, and up-regulated fibrinogen expression (42). The left atrium has been reported for its sensitivity to oxidative stress (43), and the xanthine oxidase enzyme present in the left atrium seems to boost atrial oxidative stress in patients with AF (44). In a recent experimental study, the enzymatic activity of xanthine oxidase in left atrial appendages was 4.4 times higher in the AF group compared to the controls (45). Also, documented evidence revealed that SUA can result in overloading calcium and reducing sodium channels, and exacerbating the cellular injury. These pathological processes endorse left atrial electrical remodeling (46). Besides, SUA has a direct effect on the activation of the local renin-angiotensin system, endothelial dysfunction, smooth muscle cell proliferation (47), and decreasing nitric oxide production (48). It should be noted that SUA not only promotes inflammation through the release of pro-inflammatory cytokines (37) but also via localized stimulation of the renin-angiotensin system (49). Therefore, the ROS and inflammation associated with the up-regulation of xanthine oxidase with hyperuricemia may participate in the mechanism to explain the observed association.

The development of AF as a result of elevated SUA among hypertension subjects has not been well-investigated, nor have mechanisms of such consequences been fully illuminated. The recent literature suggests a prominent hypertension risk associated with SUA, presumably because of the decrease in renal blood flow that would further stimulate urate reabsorption (45). Consequently, the higher levels of SUA may intensify the existing inflammation and vascular remodeling that result from underlying hypertension and influences the process of normal blood flow hemostasis adversely. Such physiological alterations due to high SUA levels could contribute to the activation of the renin-angiotensin system and endothelial dysfunction, which could eventually lead to AF (50) in hypertension patients. Thus, the link between SUA and risk of AF in hypertensive patients could be attributed to abnormal accumulation of SUA associated with low renal blood flow during the hypertension phase.

When we carried out a secondary analysis to investigate whether there was a possibility that the uricemic status might have influenced the predictive power of LAD values for AF in normouricemic and hyperuricemic group, the findings of the present study clarified that the estimated risk of AF associated with SUA varies depending on age groups and gender. Compared to the lowest tertile, the risk of AF was increased by two-folds in the highest tertiles of LAD and SUA in normouricemic women. Those patients with high LAD values, regardless of the hyperuricemic status, had a greater likelihood of having AF. Also, when patients were stratified by the size of LAD, SUA was associated with greater odds of AF in those patients present with large LAD but not in those patients with normal LAD, implying wide LAD tends to link more with AF patients than elevated SUA levels. Moreover, the estimated risk for AF in the highest tertile of LAD in >65 years women were lower in normouricemia compared to the hyperuricemia group. The incidence of AF normally increases with age, and similarly, the SUA levels increase with age. In males and females, the pathophysiology of AF can vary slightly. This is not extensively investigated, but in several studies, the risk factors for AF showed different strengths and qualities of association in men and women (10). From the viewpoint of our results, the assortment of such results depending on the hyperuricemic status, and size of LAD may resolve the previous conflicting results produced from other studies. Therefore, the findings can be used to guide the study designs to strictly classify AF patients based on uricemic status, and LAD size, in addition to age and gender grouping, to improve the reliability and robustness of the future studies.

### Limitation

The present study has a couple of strengths and limitations. The sample size of this study was relatively large. To our knowledge, no study investigated the interaction between the SUA and LAD in hypertension with AF in depth. Thus, it has been unknown up to now whether the interaction between elevated SUA levels and LAD amplifies the risk of AF in patients with hypertension. However, this study has several limitations. First, the cross-sectional design restricted the cause and effect relationship between the SUA/LAD and AF. Similarly, since a clear timeline of diagnosis and events are not in place, the cause and effect nature of LAD and SUA cannot be adequately determined. Second, the study involved hospitalized patients in Dalian, Northeast China, a region known for consuming seafood, therefore, the lifestyle and diet customs may significantly influence the SUA metabolism. Third, our study sample lacks national or regional representation that limits the ability to generalize the results at an international level. Thus, the results of this study may require replication for consistency from other parts of the country or other regions of the world. Fourth, our study didn't include data on left ventricular volume index and left atrial strain, and the lack of Holter monitoring for some patients in our study and the use of ECG to determine the presence of AF may negatively influence the accuracy of AF prevalence as some patients with paroxysmal AF may escape from ECG. Fifth, our study recruited only patients with hypertension therefore, further study is required to replicate and extend the results in the general population. Sixth, we were unable to rule out the influence of antihypertensive agents on our findings due to the retrospective design and ethical reasons that oppose the withdrawal of these medications. Nevertheless, in the multivariate model, we have considered antihypertensive treatment to reduce the confounding effect of antihypertensive use. Seventh, our study does not provide detailed data on the duration of AF, how long the patients have been on treatment for and how well-managed are the patients' co-morbidities (if there was any co-morbidity that did not fulfill the exclusion criteria).

## Conclusion

In conclusion, elevated SUA and LAEDD were independently associated with an increased prevalence of AF. The effect of SUA appeared most pronounced among those with AF in younger men. LAD associates independently with AF incidence in men and women alike. Those patients with high LAD values, regardless of the hyperuricemic status, had a greater likelihood of having AF. The risk of AF was increased by 2 folds in the top tertiles of LAD and SUA in normouricemic middle-aged women, suggesting that the interaction between SUA and LAD modifies the estimated risk of AF in women with hypertension. Further longitudinal studies are needed to prove whether lowering the SUA level may or may not be necessary to prevent AF in hypertension patients.

## Data Availability Statement

The datasets generated for this study are available on request to the corresponding author.

## Ethics Statement

The studies involving human participants were reviewed and approved by First Affiliated Hospital of Dalian Medical University. Written informed consent for participation was not required for this study in accordance with the national legislation and the institutional requirements.

## Author Contributions

All authors listed have made a substantial, direct and intellectual contribution to the work, and approved it for publication.

## Conflict of Interest

The authors declare that the research was conducted in the absence of any commercial or financial relationships that could be construed as a potential conflict of interest.
