# Supplementary material for: Does Serum Uric Acid Status Influence the Association Between Left Atrium Diameter and Atrial Fibrillation in Hypertension Patients?
Source: Front Cardiovasc Med. 2020 Nov 27;7:594788. doi: 10.3389/fcvm.2020.594788 (PMC7732653; doi:10.3389/fcvm.2020.594788)
Supplement: Supplementary file 1 [file Table_1.DOCX]

**Supplementary Table 1. Baseline characteristics among men participants.**

|  | **Age≤65 Years** | | | **Age >65 Years** | | |
| --- | --- | --- | --- | --- | --- | --- |
| [**Variable**](C:/Users/%E5%94%90%E9%9B%A8%E6%B7%87/AppData/Local/youdao/dict/Application/8.9.0.0/resultui/html/index.html#/javascript:;)**s** | **AF(n=222)** | **no-AF(n=2080)** | ***P*-Value** | **AF(n=324)** | **no-AF(n=2106)** | ***P*-Value** |
| Age (year) | 58.82±6.15 | 56.81±7.84 | <.001 | 74.31±5.60 | 73.63±5.93 | 0.050 |
| SBP (mm Hg) | 137.73±17.88 | 145.43±21.09 | <.001 | 141.59±20.01 | 145.31±20.58 | 0.002 |
| DBP (mm Hg) | 87.16±13.07 | 87.49±13.62 | 0.731 | 82.30±12.89 | 80.92±11.56 | 0.049 |
| DM (n/%) | 44(19.8%) | 536(25.8%) | 0.052 | 81(25.0%) | 629(29.9%) | 0.073 |
| Dyslipidemia (n/%) | 166(74.8%) | 1775(85.3%) | <.001 | 260(80.2%) | 1771(84.1%) | 0.082 |
| Cholesterol (mg/dL) | 178.41±39.89 | 179.36±39.68 | 0.735 | 45.03±10.52 | 44.50±10.46 | 0.020 |
| TG (mg/dL) | 146.90±88.17 | 168.21±127.95 | 0.016 | 115.58±60.08 | 127.42±72.85 | 0.005 |
| HDL (mg/dL) | 44.82±8.85 | 44.12±10.30 | 0.328 | 168.67±37.67 | 174.11±39.45 | 0.400 |
| LDL (mg/dL) | 102.06±26.40 | 102.02±30.45 | 0.983 | 102.59±29.73 | 102.05±29.80 | 0.762 |
| Smoking (n/%) | 92(43.0%) | 1079(55.2%) | 0.001 | 125(38.9%) | 833(42.0%) | 0.304 |
| Alcohol (n/%) | 70(33.5%) | 730(39.6%) | 0.087 | 75(24.4%) | 488(25.5%) | 0.661 |
| S[UA](C:/Users/%E5%94%90%E9%9B%A8%E6%B7%87/AppData/Local/youdao/dict/Application/8.9.0.0/resultui/html/index.html#/javascript:;) (mol/L) | 398.43±90.53 | 377.52±91.43 | 0.001 | 374.15±85.75 | 361.45±89.54 | 0.017 |
| Scr (umol/L) | 79.76±18.69 | 79.51±53.90 | 0.946 | 88.62±68.28 | 82.68±31.31 | 0.009 |
| LAEDD (mm) | 40.43±5.05 | 37.12±3.31 | <.001 | 39.93±4.81 | 37.17±3.72 | <.001 |
| Antihypertensive agent |  |  |  |  |  |  |
| ACEI/ARB (n/%) | 110(49.5%) | 1222(58.8%) | 0.008 | 178(54.9%) | 1131(53.7%) | 0.678 |
| β-blocker (n/%) | 149(67.1%) | 1147(55.1%) | 0.001 | 191(59.0%) | 957(45.4%) | <.001 |
| CCB (n/%) | 127(57.2%) | 1358(65.3%) | 0.017 | 188(58.0%) | 1320(62.7%) | 0.108 |
| Statin (n/%) | 154(69.4%) | 1639(78.8%) | 0.001 | 234(72.2%) | 1628(77.3%) | 0.044 |
| Diuretic (n/%) | 25(11.3%) | 278(13.4%) | 0.378 | 67(20.7%) | 367(17.4%) | 0.155 |

AF: Atrial fibrillation; SBP: Systolic blood pressure; DBP: [Diastolic](C:/Users/%E5%94%90%E9%9B%A8%E6%B7%87/AppData/Local/youdao/dict/Application/8.9.0.0/resultui/html/index.html#/javascript:;) [blood](C:/Users/%E5%94%90%E9%9B%A8%E6%B7%87/AppData/Local/youdao/dict/Application/8.9.0.0/resultui/html/index.html#/javascript:;) [pressure](C:/Users/%E5%94%90%E9%9B%A8%E6%B7%87/AppData/Local/youdao/dict/Application/8.9.0.0/resultui/html/index.html#/javascript:;); Scr: Serum creatinine; S[UA](C:/Users/%E5%94%90%E9%9B%A8%E6%B7%87/AppData/Local/youdao/dict/Application/8.9.0.0/resultui/html/index.html#/javascript:;): Serum uric acid; LAEDD: Left atrial end-diastolic diameter; TG: triglyceride; HDL: [High-density](C:/Users/%E5%94%90%E9%9B%A8%E6%B7%87/AppData/Local/youdao/dict/Application/8.9.0.0/resultui/html/index.html#/javascript:;) [lipoprotein](C:/Users/%E5%94%90%E9%9B%A8%E6%B7%87/AppData/Local/youdao/dict/Application/8.9.0.0/resultui/html/index.html#/javascript:;); LDL: low density lipoprotein; DM: [diabetes](C:/Users/%E5%94%90%E9%9B%A8%E6%B7%87/AppData/Local/youdao/dict/Application/8.9.0.0/resultui/html/index.html#/javascript:;) [mellitus](C:/Users/%E5%94%90%E9%9B%A8%E6%B7%87/AppData/Local/youdao/dict/Application/8.9.0.0/resultui/html/index.html#/javascript:;); ACEI: angiotensin-Converting Enzyme Inhibitors; ARB: Angiotensin-converting enzyme receptor blockers; CCB: Calcium channel blockers.

**Supplementary Table 2. Baseline characteristics among women participants.**

|  | **Age≤65 Years** | | | **Age >65 Years** | | |
| --- | --- | --- | --- | --- | --- | --- |
| [**Variable**](C:/Users/%E5%94%90%E9%9B%A8%E6%B7%87/AppData/Local/youdao/dict/Application/8.9.0.0/resultui/html/index.html#/javascript:;)**s** | **AF(n=123)** | **no-AF(n=1843)** | ***P*-Value** | **AF(n=359)** | **no-AF(n=2561)** | ***P*-Value** |
| Age (year) | 60.28±4.25 | 58.04±6.77 | <.001 | 74.96±5.82 | 73.94±5.70 | 0.002 |
| SBP (mm Hg) | 141.76±19.95 | 145.03±20.78 | 0.091 | 143.78±19.56 | 148.71±21.10 | <.001 |
| DBP (mm Hg) | 84.18±13.32 | 84.43±12.73 | 0.830 | 79.66±13.05 | 79.69±11.86 | 0.970 |
| DM (n/%) | 36(29.3%) | 462(25.1%) | 0.300 | 103(28.7%) | 875(34.2%) | 0.040 |
| Dyslipidemia (n/%) | 88(71.5%) | 1428(77.5%) | 0.129 | 279(77.7%) | 2104(82.2%) | 0.042 |
| Cholesterol (mg/dL) | 193.22±42.38 | 197.50±43.41 | 0.289 | 185.73±39.59 | 193.84±42.85 | 0.001 |
| TG (mg/dL) | 156.63±108.31 | 155.45±109.12 | 0.908 | 131.17±57.47 | 147.11±92.85 | 0.002 |
| HDL (mg/dL) | 51.24±10.67 | 50.25±11.44 | 0.354 | 49.39±10.45 | 49.92±11.22 | 0.392 |
| LDL (mg/dL) | 109.24±27.63 | 108.15±30.28 | 0.696 | 107.44±29.95 | 107.48±29.97 | 0.978 |
| Smoking (n/%) | 0(0.0%) | 25(1.5%) | 0.406 | 2(0.6%) | 52(2.1%) | 0.044 |
| Alcohol (n/%) | 0(0.0%) | 17(1.0%) | 0.623 | 0(0.0%) | 7(0.3%) | 0.606 |
| S[UA](C:/Users/%E5%94%90%E9%9B%A8%E6%B7%87/AppData/Local/youdao/dict/Application/8.9.0.0/resultui/html/index.html#/javascript:;) (mol/L) | 327.87±77.07 | 308.67±76.31 | 0.007 | 324.72±85.83 | 316.89±81.38 | 0.090 |
| Scr (umol/L) | 59.86±12.77 | 56.98±17.90 | 0.079 | 65.63±15.45 | 63.21±23.32 | 0.056 |
| LAEDD (mm) | 38.33±4.96 | 35.74±2.98 | <.001 | 39.12±5.17 | 36.27±3.37 | <.001 |
| Antihypertensive agent |  |  |  |  |  |  |
| ACEI/ARB (n/%) | 74(60.2%) | 990(53.7%) | 0.165 | 207(57.7%) | 1436(56.1%) | 0.570 |
| β-blocker (n/%) | 85(69.1%) | 884(48.0%) | <.001 | 250(69.6%) | 1223(47.8%) | <.001 |
| CCB (n/%) | 70(56.9%) | 1119(60.7%) | 0.403 | 240(66.9%) | 1693(66.1%) | 0.780 |
| Statin (n/%) | 79(64.2%) | 1312(71.2%) | 0.100 | 252(70.2%) | 1988(77.6%) | 0.002 |
| Diuretic (n/%) | 26(21.1%) | 162(8.8%) | <.001 | 61(17.0%) | 390(15.2%) | 0.387 |

AF: Atrial fibrillation; SBP: Systolic blood pressure; DBP: [Diastolic](C:/Users/%E5%94%90%E9%9B%A8%E6%B7%87/AppData/Local/youdao/dict/Application/8.9.0.0/resultui/html/index.html#/javascript:;) [blood](C:/Users/%E5%94%90%E9%9B%A8%E6%B7%87/AppData/Local/youdao/dict/Application/8.9.0.0/resultui/html/index.html#/javascript:;) [pressure](C:/Users/%E5%94%90%E9%9B%A8%E6%B7%87/AppData/Local/youdao/dict/Application/8.9.0.0/resultui/html/index.html#/javascript:;); Scr: Serum creatinine; S[UA](C:/Users/%E5%94%90%E9%9B%A8%E6%B7%87/AppData/Local/youdao/dict/Application/8.9.0.0/resultui/html/index.html#/javascript:;): Serum uric acid; LAEDD: Left atrial end-diastolic diameter; TG: triglyceride; HDL: [high-density](C:/Users/%E5%94%90%E9%9B%A8%E6%B7%87/AppData/Local/youdao/dict/Application/8.9.0.0/resultui/html/index.html#/javascript:;) [lipoprotein](C:/Users/%E5%94%90%E9%9B%A8%E6%B7%87/AppData/Local/youdao/dict/Application/8.9.0.0/resultui/html/index.html#/javascript:;); LDL: low density lipoprotein; DM: [diabetes](C:/Users/%E5%94%90%E9%9B%A8%E6%B7%87/AppData/Local/youdao/dict/Application/8.9.0.0/resultui/html/index.html#/javascript:;) [mellitus](C:/Users/%E5%94%90%E9%9B%A8%E6%B7%87/AppData/Local/youdao/dict/Application/8.9.0.0/resultui/html/index.html#/javascript:;); ACEI: angiotensin-Converting Enzyme Inhibitors; ARB: angiotensin-converting enzyme receptor blockers; CCB: calcium channel blockers.

**Supplementary Table 3. The relationship between the tertiles of SUA/ LAEDD and Atrial fibrillation**

|  | **Men** | | | | | | |
| --- | --- | --- | --- | --- | --- | --- | --- |
|  |  | **Age ≤65** | | | **Age>65** | | |
| **Models** | **Variable** | **OR** | **CI (95%)** | ***P-*Value** | **OR** | **CI (95%)** | ***P-*Value** |
| **Model 1** | SUA-T1 | 1.000 | (Ref) |  | 1.000 | (Ref) |  |
|  | SUA-T2 | 1.949 | (1.319, 2.880) | .001 | 1.130 | (0 .832, 1.536) | .434 |
|  | SUA-T3 | 2.090 | (1.405, 3.109) | <.001 | 1.159 | (0.852, 1.576) | .347 |
|  | LAEDD-T1 | 1.000 | (Ref) |  | 1.000 | (Ref) |  |
|  | LAEDD-T2 | 1.522 | (0.998, 2.322) | .051 | 1.714 | (1.205, 2.438) | .003 |
|  | LAEDD-T3 | 4.104 | (2.776, 6.069) | <.001 | 3.813 | (2.759, 5.270) | <.001 |
| **Model 2** | SUA-T1 | 1.000 | (Ref) |  | 1.000 | (Ref) |  |
|  | SUA-T2 | 1.888 | (1.295, 2.753) | .001 | 1.168 | (0.864, 1.579) | .313 |
|  | SUA-T3 | 1.907 | (1.303, 2.792) | .001 | 1.204 | (0.895, 1.620) | .220 |
|  | LAEDD-T1 | 1.000 | (Ref) |  | 1.000 | (Ref) |  |
|  | LAEDD-T2 | 1.423 | (0.944, 2.147) | .092 | 1.651 | (1.167, 2.336) | .005 |
|  | LAEDD-T3 | 3.574 | (2.456, 5.203) | <.001 | 3.610 | (2.630, 4.954) | <.001 |
|  | **Women** | | | | | | |
|  |  | **Age ≤65** | | | **Age>65** | | |
|  | **Variable** | **OR** | **CI (95%)** | ***P-*Value** | **OR** | **CI (95%)** | ***P-*Value** |
| **Model 1** | SUA-T1 | 1.000 | (Ref) |  | 1.000 | (Ref) |  |
|  | SUA-T2 | 1.492 | (0 .903, 2.466) | 0.118 | 0.845 | (0 .630, 1.134) | .262 |
|  | SUA-T3 | 1.708 | (1.039, 2.808) | 0.035 | 1.018 | (0 .767, 1.352) | .899 |
|  | LAEDD-T1 | 1.000 | (Ref) |  | 1.000 | (Ref) |  |
|  | LAEDD-T2 | 1.146 | (0.661, 1.987) | 0.628 | 1.015 | (0.729, 1.412) | .930 |
|  | LAEDD-T3 | 2.655 | (1.664, 4.236) | <.001 | 3.190 | (2.396, 4.246) | <.001 |
| **Model 2** | SUA-T1 | 1.000 | (Ref) |  | 1.000 | (Ref) |  |
|  | SUA-T2 | 1.515 | (0.919, 2.498) | 0.103 | 0.875 | (0.656, 1.167) | .364 |
|  | SUA-T3 | 1.687 | (1.033, 2.755) | 0.037 | 1.077 | (0.820, 1.414) | .595 |
|  | LAEDD-T1 | 1.000 | (Ref) |  | 1.000 | (Ref) |  |
|  | LAEDD-T2 | 1.135 | (0.656, 1.963) | 0.651 | 0.965 | (0.695, 1.340) | .831 |
|  | LAEDD-T3 | 2.478 | (1.563, 3.928) | <.001 | 2.846 | (2.153, 3.763) | <.001 |

Note: Model 1: adjusted for age and Model 2 adjusted for age, SBP, serum creatinine, and smoking
